# Specific modulation of corticomuscular coherence during submaximal voluntary isometric, shortening and lengthening contractions

**DOI:** 10.1038/s41598-021-85851-w

**Published:** 2021-03-18

**Authors:** Dorian Glories, Mathias Soulhol, David Amarantini, Julien Duclay

**Affiliations:** 1 ToNIC, Université de Toulouse, Inserm, UPS, Toulouse, France; 2 Faculty of Sport Science, University Paul Sabatier, Toulouse, France

**Keywords:** Motor cortex, Spinal cord

## Abstract

During voluntary contractions, corticomuscular coherence (CMC) is thought to reflect a mutual interaction between cortical and muscle oscillatory activities, respectively measured by electroencephalography (EEG) and electromyography (EMG). However, it remains unclear whether CMC modulation would depend on the contribution of neural mechanisms acting at the spinal level. To this purpose, modulations of CMC were compared during submaximal isometric, shortening and lengthening contractions of the soleus (SOL) and the medial gastrocnemius (MG) with a concurrent analysis of changes in spinal excitability that may be reduced during lengthening contractions. Submaximal contractions intensity was set at 50% of the maximal SOL EMG activity. CMC was computed in the time–frequency domain between the Cz EEG electrode signal and the unrectified SOL or MG EMG signal. Spinal excitability was quantified through normalized Hoffmann (H) reflex amplitude. The results indicate that beta-band CMC and normalized H-reflex were significantly lower in SOL during lengthening compared with isometric contractions, but were similar in MG for all three muscle contraction types. Collectively, these results highlight an effect of contraction type on beta-band CMC, although it may differ between agonist synergist muscles. These novel findings also provide new evidence that beta-band CMC modulation may involve spinal regulatory mechanisms.

## Introduction

The control strategy employed by the nervous system during voluntary contractions can be investigated through the analysis of neural interactions between the brain and the contracting muscle. Such interactions have been quantified by computing corticomuscular coherence (CMC) between electroencephalography (EEG) and surface electromyography (EMG) oscillatory activities^1^. CMC has been detected between the primary motor cortex (M1) and muscle activities during isometric contractions, which is why it is considered to directly reflect a cortical regulation process occurring in the motor system via the corticospinal pathway^2–7^. While previous studies assumed CMC to be the result of the mutual interaction between the M1 and contracting muscles via descending motor pathways and ascending somatosensory pathways^8^, the neural mechanisms involved in CMC modulation are yet to be clarified. It has been suggested that corticomuscular interactions could be modulated at the spinal level^9^. Consistent with this assumption, Matsuya et al. (2017)^10^ recently provided evidence that the amount of recurrent inhibition, a spinal inhibitory mechanism, was significantly linearly related to the magnitude of CMC, and showed that greater recurrent inhibition was associated with weaker CMC magnitude. These authors concluded that the modulation by Renshaw cell activity works to weaken oscillations issued from the cortex, which would imply a “neural filter” function of local spinal loops on cortico-spinal oscillations.

However, while these results were obtained during isometric contractions, it has since been shown that the amount of recurrent inhibition increases solely during lengthening when compared with isometric and shortening contractions^11,12^. Added to the fact that, in most cases, the discharge rate of motor units decreases during lengthening compared with shortening contractions^13,14^, these findings suggest that muscle oscillatory activity may differ according to the muscle contraction type, which could result in the modulation of the neural interactions shown by CMC analysis, especially during lengthening contractions. Indeed, variations of the neural control between contraction types has been largely explored using different approaches, with the conclusion that the control strategy employed by the nervous system to activate muscles differs during lengthening contractions when compared to isometric and shortening ones (for a review, see^15^). More precisely, a specific depression of the corticospinal excitability, depending mainly on pre- and post-synaptic inhibitory mechanisms acting at the spinal level, was found during lengthening contractions in various studies^16–20^. Therefore, while most of the findings published in the literature concerning CMC are currently obtained during isometric contractions, and while only a few investigated CMC during multi-joint compound movements^21,22^, we profited from these differences in neural drive between muscle contraction types to obtain a more complete picture of the neural mechanisms involved in the modulation of CMC.

The aim of the present study was to highlight the relative contribution of neural mechanisms acting at the spinal level on CMC by comparing changes in CMC and spinal excitability during isometric, shortening and lengthening contractions of the plantar flexors. The unique neural drive strategy employed during lengthening contractions^15^ provided us with the opportunity to investigate a potential spinal modulation of CMC. Because Matsuya et al. (2017)^10^ showed that the amplitude of CMC decreased while recurrent inhibition increased and Barrué-Belou et al. (2018)^11^ observed greater recurrent inhibition during lengthening contractions, we hypothesized that CMC would be lower during lengthening contractions compared with isometric and shortening contractions.

## Results

### Study summary

**Figure 1:**
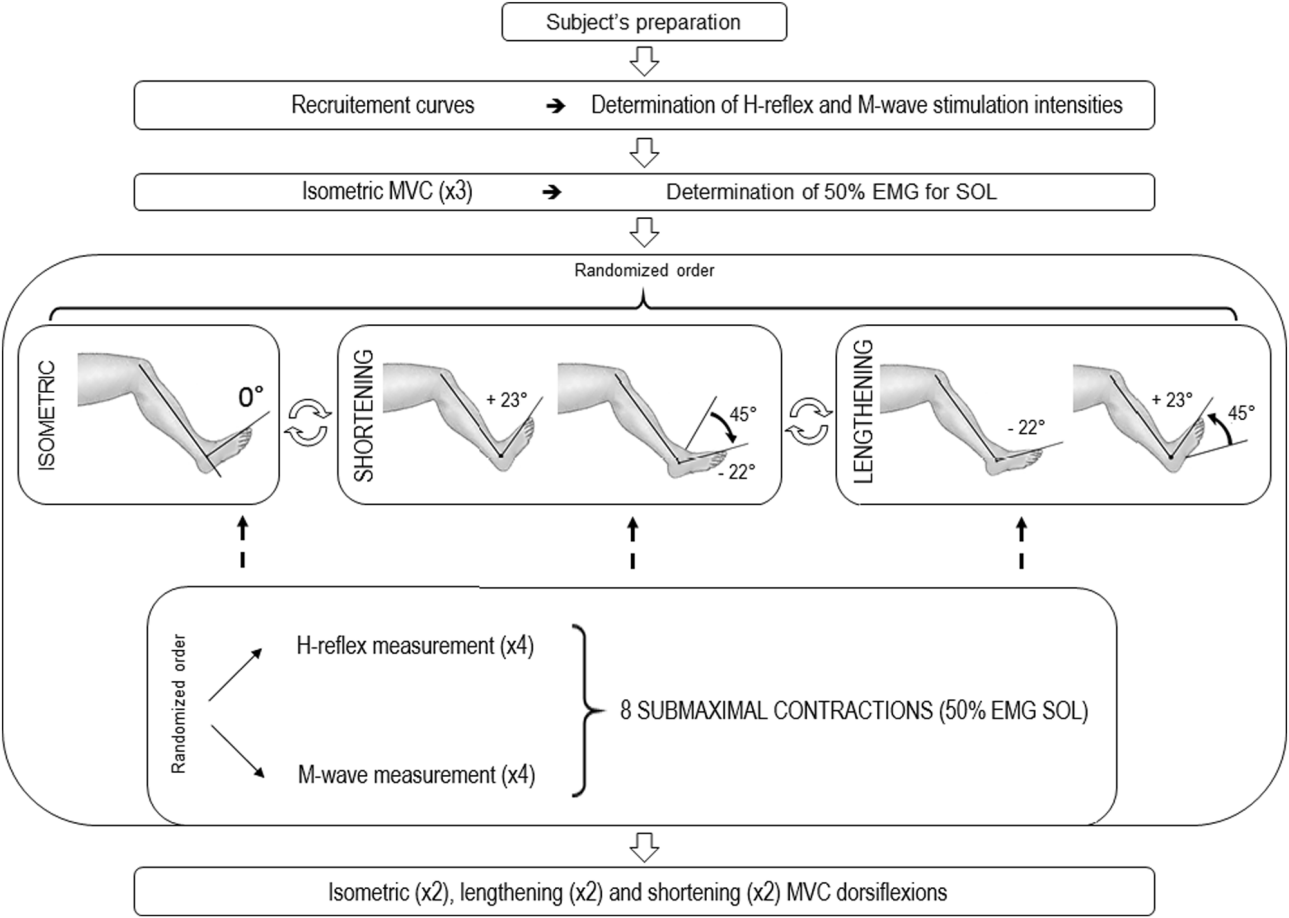
Experimental protocol. *MVC* maximal voluntary contraction, *50% EMG SOL* contraction intensity corresponding to 50% of the EMG obtained during maximal voluntary isometric contractions.

For each subject, a single experimental session, comprised of at least 24 sub-maximal contractions, was performed over a 2-h period (including set-up time). The subject was seated on an isokinetic ergometer and was asked to perform sub-maximal isometric, lengthening and shortening contractions while the EEG signal as well as soleus (SOL) and medial gastrocnemius (MG) EMG activities were recorded during every contraction in order to compute CMC. Meanwhile, nerve stimulations were needed to evoke both an H-reflex and M-wave in order to investigate changes in spinal excitability between muscle contraction types. The different steps of the experiment protocol have been summarized in Fig. 1.

### Torque and EMG activity

A significant effect of contraction type was found on the mean torque produced (F (2,48) = 72.9; p < 0.001; η^2^ = 0.75; f = 1.74). The torque produced during shortening contractions was significantly lower than during both isometric (p < 0.001) and lengthening (p < 0.001) contractions. No difference was observed between the isometric and lengthening torque (p = 0.73) (Table 1). The mean torque exerted during shortening contractions was 47.2 ± 19.2% and 49.2 ± 15.9% lower than during lengthening and isometric contractions respectively. In contrast, the SOL EMG activity (RMS/M_sup_ ratio) was similar between lengthening, isometric and shortening contractions (F (2,48) = 1.53; p = 0.23) for an equal amount of coactivation (mean value of 4.46% ± 2.63) for all contraction types (F (2,46) = 0.334; p = 0.72). During the submaximal contractions, the percentage of the respective SOL and MG maximal EMG activity was similar in both muscles (F (1,74) = 0.313; p = 0.578) (46.3 ± 6.5 and 45.6 ± 10.3%, respectively). Similarly, no effect of the contraction type was observed on the MG EMG activity (F (2,26 = 2.24; p = 0.13) (Table 1). No significant effect of the contraction type was found in the SOL (F (2,48) = 0.31; p > 0.05) or in the MG (F (2,48) = 1.08; p > 0.05) EMG mean frequency (32.5 ± 1.4 and 31.3 ± 1.8 Hz, respectively).

**Table 1:** Effect of contraction type on torque, coactivation, plantar flexors EMG activity, and on amplitudes of soleus (SOL) and medial gastrocnemius (MG) evoked potentials. Data are means ± SD. *p < 0.001: shortening vs isometric and lengthening.

Contraction type	Lengthening	Isometric	Shortening
Torque (N.m)	59.32 ± 18.4	61.52 ± 21.9	31.45 ± 14.8*
Coactivation TA (%)	4.67 ± 2.75	4.41 ± 2.57	4.30 ± 2.66
**SOL (n = 25)**			
RMS/M_sup_ (a.u.)	0.014 ± 0.007	0.013 ± 0.006	0.014 ± 0.007
M_sup_ (mV)	7.21 ± 2.55	7.43 ± 2.99	7.27 ± 2.89
MH/M_sup_ (a.u.)	0.08 ± 0.05	0.07 ± 0.03	0.07 ± 0.03
**MG (n = 14)**			
RMS/M_sup_ (a.u.)	0.011 ± 0.005	0.009 ± 0.004	0.009 ± 0.004
M_sup_ (mV)	5.62 ± 2.39	6.00 ± 2.87	5.35 ± 2.13
MH/M_sup_ (a.u.)	0.18 ± 0.18	0.19 ± 0.21	0.17 ± 0.19

### H-reflex and M-wave

**Figure 2:**
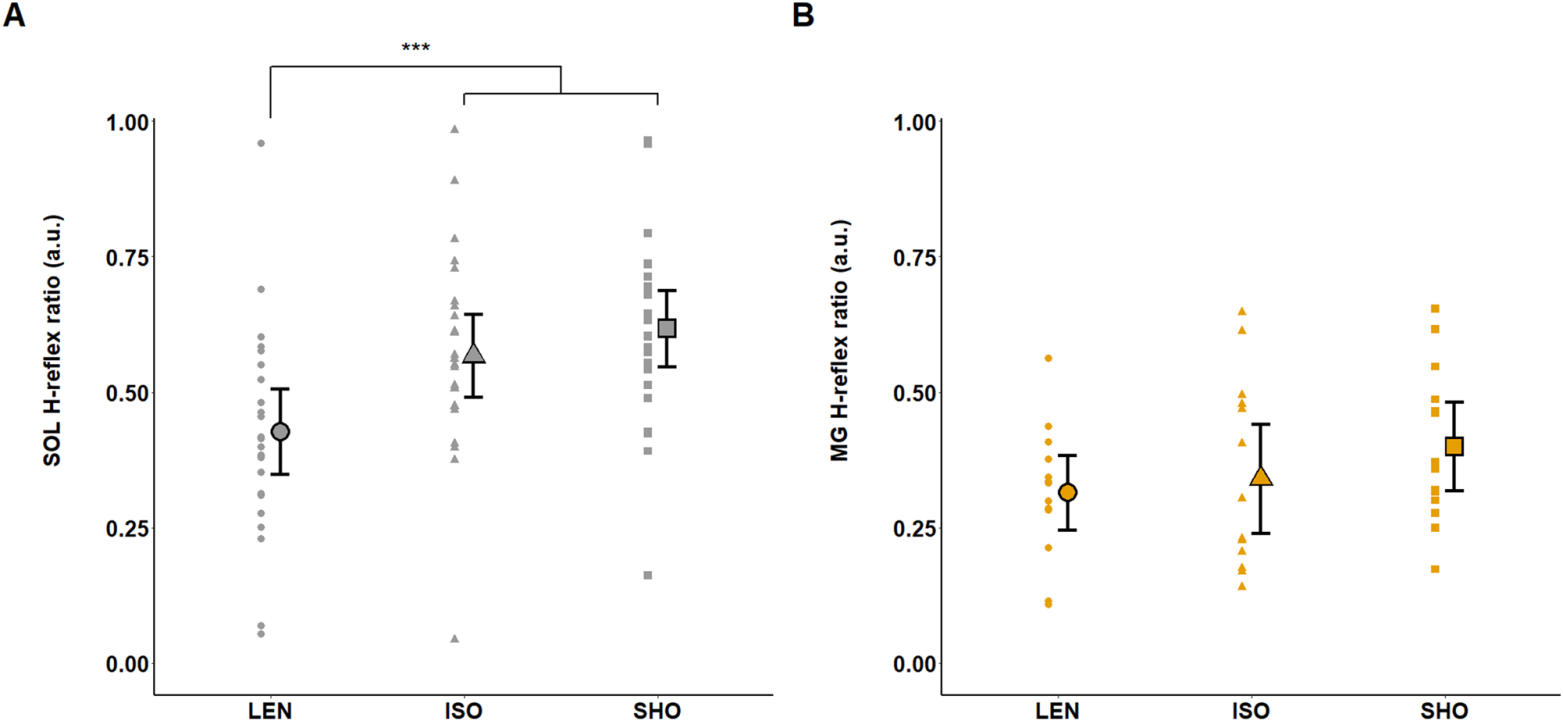
Effects of muscle contraction type on the soleus and the medialis gastrocnemius spinal excitability. Modulations of spinal excitability are computed with the muscle H-reflex (H_sup_) normalized to the corresponding M-wave (M_sup_) amplitudes, in arbitrary units (a.u). Modulations of the H_sup_/M_sup_ ratio for (**A**) the soleus (n = 25) and (**B**) the medialis gastrocnemius (n = 14) are shown during lengthening (LEN), isometric (ISO) and shortening (SHO) contractions. Error bars represent the 95% CI of the mean. Lengthening vs isometric and shortening: ***p < 0.001.

A significant effect of contraction type was found on the SOL H-reflex normalized by the corresponding M-wave (H_sup_/M_sup_ ratio) (F (2,48) = 27.5; p < 0.001; η^2^ = 0.53; f = 1.07). The SOL H_sup_/M_sup_ ratio was significantly lower during lengthening compared to isometric (p < 0.001) and shortening (p < 0.001) contractions by 13.8 ± 11.3% and 20.4 ± 15.1% respectively. No significant difference was observed for this ratio between isometric and shortening contractions (p = 0.25) (Fig. 2A). In contrast, no significant difference between contraction types was observed for MG H_sup_/M_sup_ ratios (F (2,26) = 1.63; p = 0.22) (Fig. 2B). The maximal M-wave (M_sup_) was similar regardless of the muscle contraction type for the SOL (F (2,48) = 0.81; p = 0.77) and the MG (F (2,26) = 1.40; p = 0.27). The MH_sup_/M_sup_ ratios were not significantly affected by contraction type for the SOL (mean value of 0.07 ± 0.04) (F (2,48) = 1.01; p = 0.37) and the MG (mean value of 0.18 ± 0.19) (F (2,26) = 0.25; p = 0.78) (Table 1).

### Corticomuscular coherence

**Figure 3:**
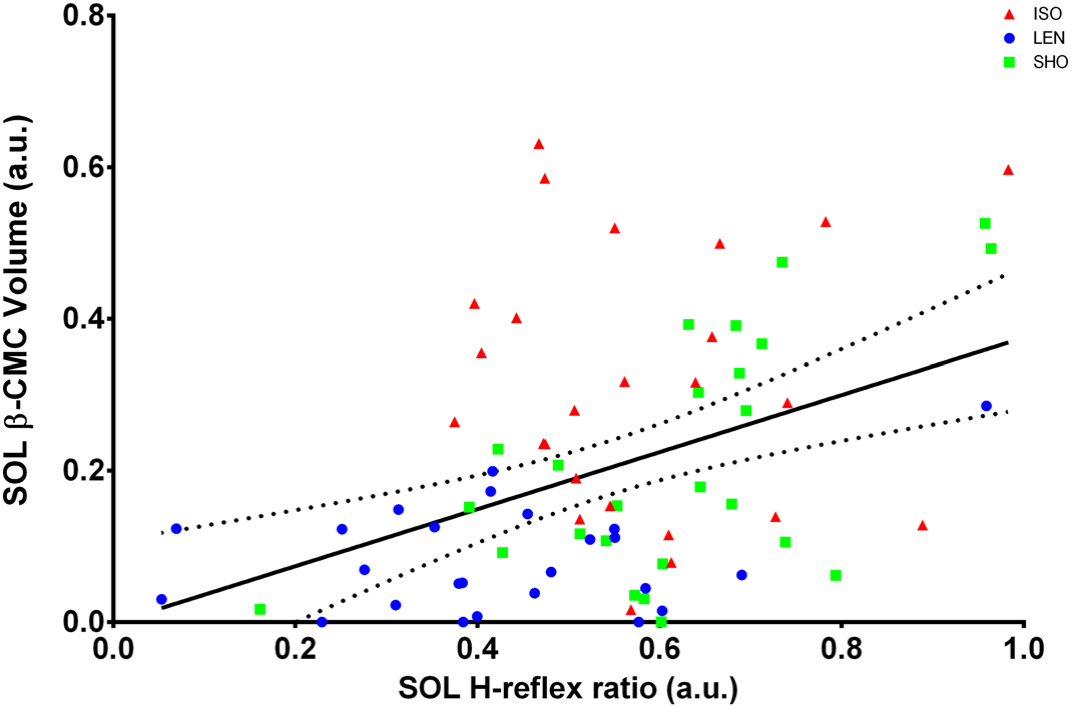
Correlation between beta-band CMC and normalized H-reflex in the SOL. The relationship between beta-band CMC (SOL β-CMC Volume) and the normalized H-reflex (SOL H-reflex ratio) have been calculated in the SOL, across all participants. A significant correlation was observed between SOL β-CMC Volume and SOL H-reflex ratio (r = 0.421, p < 0.001). Data obtained during lengthening contractions (LEN) are shown in blue, during isometric contractions (ISO) in red and during shortening ones (SHO) in green. The solid line shows the estimated regression line, and the dashed lines represent its 95% CI.

**Figure 4:**
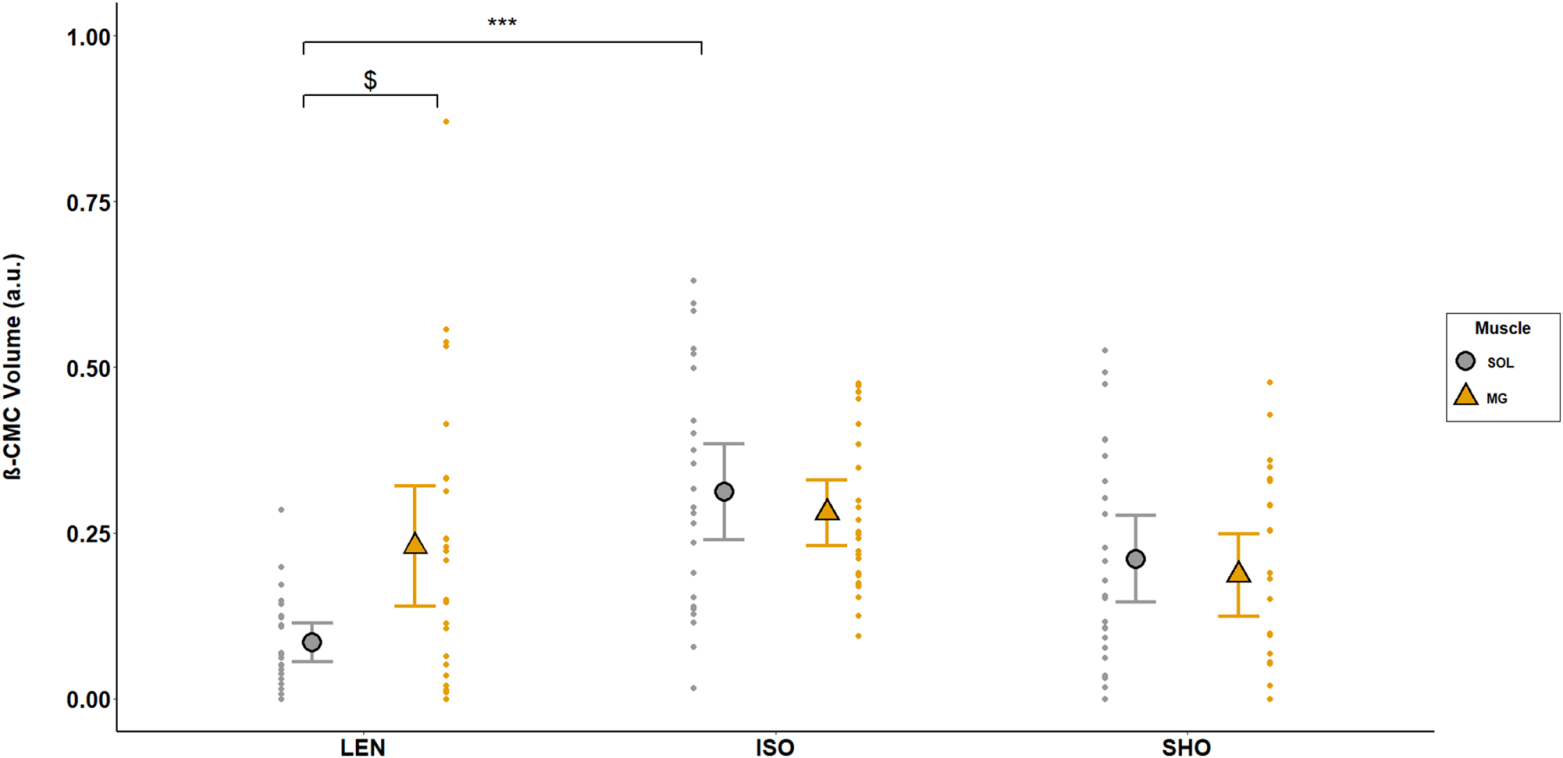
Effects of muscle contraction type and muscle type on beta-band CMC. CMC was computed as the volume in the beta-band (13–32 Hz) during the last 200 ms prior to the neurostimulation onset. Modulations are shown in the soleus (grey circles) and the medialis gastrocnemius (yellow triangles) during lengthening (LEN), isometric (ISO) and shortening (SHO) contractions. Error bars represent the 95% CI of the mean (n = 25). Lengthening vs isometric in SOL: ***p < 0.001; SOL vs MG during lengthening: ^$^p < 0.01.

Repeated measures multivariate ANOVA revealed a statistically significant interaction between contraction type and muscle for CMC computed in the beta-band (β-CMC) (F (2,48) = 5.85; p = 0.003; η^2^ = 0.212; f = 0.52). β-CMC computed between the SOL and the Cz electrode (β-CMC_SOL-Cz_) was significantly lower during lengthening contractions compared to isometric contractions (p < 0.001). This reduction (ΔCMC) was not correlated to the reduction in the SOL H_sup_/M_sup_ observed between lengthening and isometric contractions (ΔH_sup_/M_sup_) (p = 0.28). However, in the SOL, β-CMC was positively correlated with H_sup_/M_sup_ (r = 0.42; p < 0.001) (Fig. 3). No significant difference was found in β-CMC_SOL-Cz_ between isometric and shortening contractions (p = 0.38). β-CMC_SOL-Cz_ was significantly lower than β-CMC computed between the MG and the Cz electrode (β-CMC_MG-Cz_) during lengthening contractions (p = 0.026). No significant effect of contraction type was shown for β-CMC_MG-Cz_ (p > 0.05) (Fig. 4).

**Figure 5:**
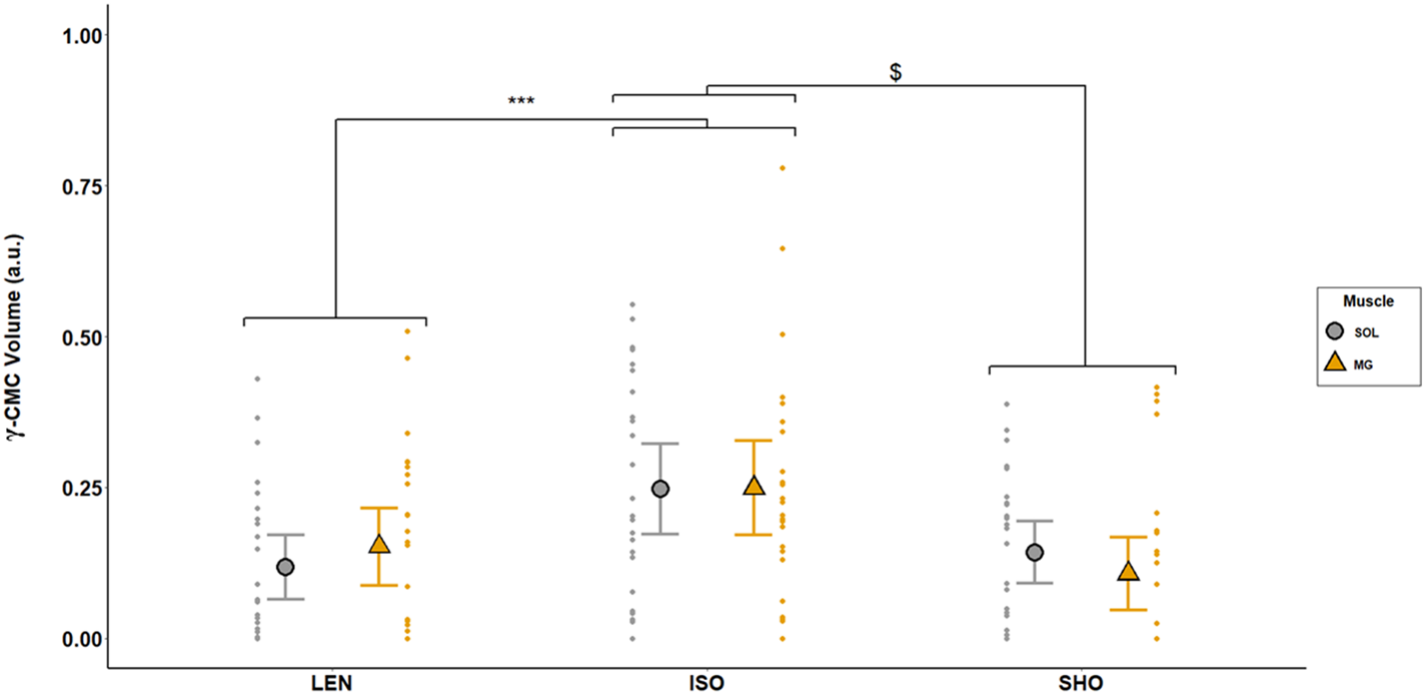
Effects of muscle contraction type on gamma-band CMC. CMC was computed as the volume in the gamma-band (33–52 Hz) during the last 200 ms prior to the neurostimulation onset. Modulations are shown in the soleus (grey circles) and the medialis gastrocnemius (yellow triangles) during lengthening (LEN), isometric (ISO) and shortening (SHO) contractions. Error bars represent the 95% CI of the mean (n = 25). Lengthening vs isometric: ***p < 0.01; shortening vs isometric: ^$^p < 0.01.

A significant effect of contraction type was found on gamma-band CMC (F (2,48) = 8.06; p < 0.001; η^2^ = 0.251; f = 0.58), which was significantly lower during both lengthening (p = 0.007) and shortening (p = 0.003) contractions compared to isometric contractions for both the SOL and the MG (Fig. 5). Gamma-band CMC was similar between the two muscles (F (1,24) = 8e−5; p = 0.99).

## Discussion

In order to deepen our knowledge of the neural mechanisms that regulate corticomuscular interactions, the present study investigated variations in CMC and spinal excitability in two synergist plantar flexor muscles during isometric, lengthening and shortening contractions. The main finding of the present study was a significant effect of contraction type on the CMC amount in the beta band for the SOL muscle, with a decrease observed during lengthening compared to isometric contractions. Meanwhile, a decrease in spinal excitability was also observed in the SOL during lengthening compared to isometric and shortening contractions. However, the effect of contraction type on CMC amount in the beta band seems to be modulated differently depending on synergistic muscles.

### Corticomuscular coherence is specifically modulated during lengthening contractions

While most of the previous studies investigated CMC during isometric contractions^3,6,23–27^, the current study was the first designed to assess CMC during both isokinetic lengthening and shortening contractions compared to isometric contractions. Significant corticomuscular coupling during both isometric and anisometric contractions was observed, yet CMC quantified at the Cz electrode in the beta-band was lower during lengthening contractions compared to isometric contractions for the SOL muscle, whereas no difference was obtained between isometric and shortening contractions. Considering that beta-band CMC is thought to reflect a mutual interaction between the M1 and the contracting muscle via corticospinal pathways during isometric contractions^2,8,23,28^, the observed muscle contraction type effect on beta-band CMC strengthens the fact that the neural activation strategy might be specific during lengthening contractions (for a review see^15^).

This specifically lower beta-band CMC during lengthening contractions may be the consequence of differences in torque produced by the subject between contraction types because it has been suggested that an increase in torque during isometric contractions could lower beta-band CMC^26,27^, although it may depend on the muscle investigated^29^. In the current study, a visual feedback of the RMS value of the SOL EMG was used to ensure that its activity did not differ according to the contraction type. Therefore, for similar SOL EMG activity, a significant effect of the contraction type was shown on plantar flexors torque. Thus, beta-band CMC changes observed in the current study for the SOL could result from changes in torque, independently from contraction type effect. However, no difference was reported in the current study in beta-band CMC between isometric and shortening contractions while torque was lower during shortening compared to isometric contractions. Moreover, lower beta-band CMC was found in the SOL during lengthening contractions compared with isometric contractions while torque did not differ significantly between these two contraction types. Although further investigations into the influence of muscle contraction intensity on beta-band CMC during anisometric contractions are needed to make a definite conclusion, these findings strongly indicate that the differences in torque could not explain the modulation of beta-band CMC when muscle contraction types are compared. The lower beta-band CMC observed in the current study during lengthening contractions could also result from a disruption of the EMG–EEG frequency coupling, since an increase of the EMG mean frequency was previously shown during anisometric maximal voluntary contractions (MVCs) compared to isometric ones^30^. However, the SOL EMG mean frequency was similar between contraction type in the current study suggesting that the lower CMC observed during lengthening is unlikely to be explained by a shift of the SOL EMG mean frequency.

Furthermore, beta-band CMC does not only assess a descending oscillatory coupling from the M1 to the muscle, as ascending afferent drives are essential to the establishment of corticomuscular interactions^8,31^. Beta-band CMC has been initially reported to be reduced during movements, due to differences in somatosensory afference integration^32^. However, more recent studies whose aim was to investigate CMC modulation between isometric and dynamic contractions reported that beta band CMC may be similar between isometric and anisometric contractions^33–35^. Accordingly, no difference in beta-band CMC between isometric and shortening contractions in the SOL was observed in the current study suggesting that differences in somatosensory afference integration between isometric and anisometric contractions do not seem to mainly explain the lower beta-band CMC found in the SOL during lengthening contractions either. Furthermore, a lower CMC magnitude was shown after a decreased somatosensory afferent feedback obtained with a local deafferentation induced by ischemia^36^. However, lengthening contractions are rather associated with an increase in afferent input through muscle spindles, compared to isometric contractions^37,38^. Consequently, although we cannot completely rule out a direct influence of possible changes in the muscle oscillatory feedback between contraction types on CMC, lower beta-band CMC found in the SOL during lengthening contractions does not seem to be linked to greater afferent input. Otherwise, this increase in sensory feedback during lengthening contractions could result in higher gamma-band CMC (33–52 Hz) that is thought to reflect mechanisms underlying the integration of task related cortical components during sensorimotor tasks and proprioceptive feedback^39,40^. Accordingly, previous research groups have reported that CMC may ‘shift’ to higher frequencies in the frequency-domain during sinusoidal modulation of the force production during weak isometric contractions^41^ or in the time–frequency domain during shortening isokinetic contractions^34^. In contrast, gamma-band CMC was lower in the current study during both lengthening and shortening contractions compared to isometric ones, for both the SOL and the MG. Therefore, even though our results point toward a specific regulation of gamma-band CMC during anisometric contractions, we did not find any increase in gamma-band CMC during lengthening contractions that could explain, due to a ‘shift’ in the CMC frequency from beta- to gamma-band, the lower amount of beta-band CMC observed for the SOL during lengthening contractions compared to isometric ones. This discrepancy with the findings of certain studies^33–35,41^ could be due on the one hand to the fact that CMC is task related^6^. Indeed, contrary to previous studies that involved weaker contractions^34,41^ and/or multi-joint compound movements^33,35^, the current study was the first to analytically investigate the muscle contraction type effect during isokinetic contractions with stronger contractions. On the other hand, while the method we used for CMC processing rendered a meaningful interpretation of synchronization processes with an excellent balance between the localization of time and frequency^42^, it may also induce differences with previous studies. In spite of these methodological concerns, we are confident that, in the current study, the decrease in beta-band CMC during lengthening contractions for the SOL is not related to a ‘shift’ in the CMC frequency from the beta- to the gamma-band.

### Beta-band corticomuscular coherence is modulated by spinal inhibitory mechanisms

Furthermore, it was recently suggested that beta-band CMC could be modulated locally at the spinal level^9,10^ and may not only derive from direct descending efferent and ascending afferent drive interactions. Indeed, neural oscillations may be ‘filtered’ by inhibitory mechanisms acting at the spinal level, which could partly regulate corticomuscular oscillatory interactions^9^. The concurrent comparison of beta-band CMC and spinal excitability between contraction types offered us a paradigm where spinal excitability could be modulated in order to study its influence on the modulation of corticomuscular interactions. Consequently, the current study provided us with the opportunity to investigate this so-called ‘spinal filter’, knowing that the decrease in corticospinal excitability observed during lengthening contractions depends mainly on presynaptic and postsynaptic inhibitory mechanisms acting at the spinal level^19,20^. This decrease in spinal excitability during lengthening contractions was also observed in the current study as shown by the lower SOL H_sup_/M_sup_ ratio during lengthening compared to isometric and shortening contractions and was for the first time associated to a concurrent decrease in the SOL beta-band CMC during lengthening compared to isometric contractions. Even though no linear correlation was found between these variations, the positive linear correlation between the magnitude in beta-band CMC and the H_sup_/M_sup_ further highlights the association between corticomuscular interactions and spinal excitability. Moreover, the similar behaviour of CMC and the spinal excitability between contraction types strongly supports previous studies reporting that the beta-band CMC modulation may involve spinal regulatory mechanisms^9,10^, and underpins that this spinal filter may be enhanced during lengthening contractions for the SOL. This assumption is further strengthened when the effect of muscle contraction type on beta-band CMC is compared between the SOL and the MG. As classically described^19,20^, the MG normalized H-reflex is not reduced during lengthening contractions in a subgroup of subjects in which simulation conditions allowed its study (n = 14) thus reinforcing the proposal that this muscle is less sensitive to spinal inhibitory mechanisms than the SOL. This difference in regulation of spinal excitability between the SOL and the MG may be associated with the greater beta-band CMC amount observed in the MG compared with the SOL during lengthening contractions and the lack of contraction type effect on beta-band CMC for the MG muscle. Although it was previously reported that beta-band CMC amount may differ between muscles^29^, the beta-band CMC between the SOL and the MG in the current study was similar during isometric and shortening contractions, while their respective contribution to the task was similar (46.3 ± 6.5 and 45.6 ± 10.3% of their maximal EMG activity, respectively). Hence, this result indicates that the difference of beta-band CMC observed between these two muscles during lengthening contractions is unlikely to be explained by an intrinsic difference of beta-band CMC between them. While our data does not shed light on the precise regulation mechanisms, the comparison of beta-band CMC and H-reflex modulations between the SOL and the MG confirms the hypothesis that spinal inhibitory mechanisms may act as beta-band CMC regulatory mechanisms.

Amongst several neural inhibitory mechanisms involved in spinal excitability regulation, Matsuya and co-workers (2017)^10^ suggested that the recurrent inhibition generated by spinal Renshaw cells might work to ‘filter’ neural oscillations due to its functional role in the modulation of both spatial and temporal pattern of motoneuronal activity^43^ and thus modulate CMC. One of the main findings from this study was that greater recurrent inhibition was associated with weaker beta-band CMC during isometric contractions. Hence, the higher recurrent inhibition found during lengthening contractions compared to isometric contractions^11,12^ might explain the lower beta-band CMC observed in our study during lengthening contractions. Consequently, although further investigations into the effect of recurrent inhibition on CMC during anisometric contractions are needed to make a definite conclusion, beta-band CMC in the SOL might be lower during lengthening contractions because of disrupted oscillatory muscle activation induced by the higher recurrent inhibition generated at the spinal level.

## Conclusion

In conclusion, while most of the studies from the literature limited their investigations of changes in CMC to isometric contractions, the current study highlights an effect of contraction type on both beta-band and gamma-band CMC during plantar flexions, which should be taken into consideration in future studies on CMC modulations during complete physiological movements involving different muscle contraction types. Gamma-band CMC was lower during shortening and lengthening contractions compared to isometric ones, which demonstrates a specific modulation of high frequency corticomuscular interactions during anisometric contractions. Moreover, the concurrent changes in beta-band CMC and spinal excitability in both the SOL and the MG provide strong new evidence that neural mechanisms acting at the spinal level modulate beta-band CMC, although their action may differ between synergist muscles.

## Material and method

### Subjects

Twenty-five healthy volunteers [age: 25.6 ± 5.9 years, height: 173.1 ± 7.5 cm, weight: 70.6 ± 11.5 kg, means ± standard deviation (SD)] with no history of neurological injuries or diseases participated in this study. All subjects signed an informed consent form after receiving explicit information about the experimental design. All experimental protocols were approved by the local ethic committee of the Faculty of Sport Sciences and Human Movement at Paul Sabatier University (Toulouse 3) in Toulouse, France. All procedures used in this study were conformed with the Declaration of Helsinki, except for registration in a public database.

### Measurements

#### Mechanical data

Ankle joint torque produced during voluntary plantar flexion was measured using a calibrated isokinetic dynamometer (Biodex S4, Shirley, NY) while subjects were seated with the hip and knee joints both flexed, at 80° (0° = anatomical position) and 60° (0° = full extension) respectively. Data was obtained only from the right foot, which was tightly attached to the dynamometer’s footplate accessory. The ankle joint was aligned with the dynamometer’s motor axis. Subjects were fastened to the chair by two shoulder harnesses and one abdominal harness to minimize the body’s movement and to limit the contribution of other muscle groups aside from ankle plantar- and dorsi-flexors in net torque production. Particular care was taken in avoiding head movements, and a neck brace was used to reduce the influence of neck muscle electrical activity on the EEG signal. Net torque and angular position were recorded at 2 kHz using a Biopac MP150 system and Acknowledge software (Biopac Systems, Santa Barbara, CA, USA). The “baseline” torque produced by the limb weight plus accessories was systematically subtracted from all active torque signals.

#### Electroencephalography

The EEG signal was recorded at 1024 Hz by a 64-channel ActiveTwo system (BioSemi, Amsterdam, The Netherlands). The EEG electrode locations followed the 10–20 international system. The EEG electrodes impedances were kept below 20 kΩ. To minimize contamination of the EEG signal, all subjects were asked to relax the upper body while performing sub-maximal contractions.

#### Electromyography

The EMG activities of the right side SOL, MG and tibialis anterior (TA) muscles were recorded by pairs of 8 mm diameter silver-silver chloride surface electrodes (inter-electrode distance: 2 cm) and sampled at 5 kHz using a Biopac MP150 system and Acknowledge software (Biopac Systems, Santa Barbara, CA, USA; common mode rejection ratio CMRR > 110 dB, gain: 1000, bandwidth: 10–500 Hz). After preparing the skin to reduce impedance below 5 kΩ, SOL, MG and TA electrodes were placed in accordance with the SENIAM recommendations^44^. The SOL and MG electrodes were thus fixed lengthwise over the middle of their respective muscle bellies. If needed, the placements were adjusted with the aim of obtaining the greatest M-wave and H-reflex amplitudes in response to tibial nerve stimulation. Because spinal excitability can be affected by antagonist muscle activity, the EMG activity of the TA was also recorded, in order to ensure that no difference in coactivation that could influence the spinal excitability would occur between contraction types^45^. The TA electrodes were positioned one-third of the way between the proximal fibula tip and medial malleolus. The reference electrode was placed on the patella on the left leg.

#### Nerve stimulation

Rectangular electrical stimuli were delivered to the posterior tibial nerve to evoke both an H-reflex and an M-wave responses in the SOL and the MG (pulse duration: 1 ms; Digitimer stimulator, model DS7, Hertfordshire, UK). The cathode (8-mm-diameter silver disk electrodes) was placed in the popliteal fossa and an adhesive anode (10 × 5 cm, Medicompex SA, Ecublens, Switzerland) was placed on the patellar tendon. The stimulation site providing the greatest amplitude for the evoked response was first located by a hand-held cathode ball electrode (0.5-cm diameter). Once found, the stimulation electrode was firmly fixed to this site with straps and tape.

### Experimental design

Firstly, the subjects performed three 4-s plantar flexors maximal voluntary isometric contractions, with 1-min rest intervals. The RMS values of the SOL EMG over a 500-ms period when the torque plateaued were computed and averaged across trials. Half of this EMG activity (50% EMG contraction) was then used to set the submaximal contraction intensity required during the experiments. Visual feedback of the RMS value of the SOL EMG activity (EMG_SOL_) (computed online with an integration time of 250 ms) was displayed on a monitor screen 150 cm in front of the subjects. For each submaximal contraction, subjects were asked to reach the target and maintain it throughout the entire duration of the contraction. The isometric contractions were performed at 0° (corresponding to an ankle angle of 90°) and the stimulations were triggered manually when the torque plateaued. We made sure to wait for the subject to reach a steady state of contraction (i.e., between 1.5 and 2 s after the subject had reached the target) to avoid CMC reorganisation induced by a transition between sensorimotor states^46^. The shortening and lengthening contractions measurements were taken at 30°·s^−1^ constant velocity over a 45° ankle range of motion from + 23° (plantarflexion) to − 22° (dorsiflexion). During the anisometric conditions, each contraction began with an isometric preactivation of ~ 3 s. A stimulation was always automatically delivered when the joint angle passed 0° to avoid changes in either the M-wave or the H-reflex size due to variation in muscle length^47^.

H-reflex recruitment curves were obtained at rest to carefully determine the stimulus intensities necessary to induce maximal SOL H-reflex and maximal SOL M-wave. The intensity corresponding to the maximal M-wave response was multiplied by 1.5 to obtain the intensity used to elicit the M-wave during contractions (M_sup_). Thereafter, both intensities were used during the shortening and the lengthening contractions. Then, subjects were asked to perform eight submaximal contractions (50% EMG contraction) of the plantar flexors per contraction type with 20 s rest intervals. The contraction type order was randomized. For each contraction type, two different sequences were carried out in a random order. The first sequence being four contractions superimposed with the H-reflex stimulus intensity to evoke a superimposed H-reflex (H_sup_). The second being four contractions superimposed with the M-wave stimulus intensity to evoke a superimposed M-wave (M_sup_). An H-reflex measurement, normalized with M-wave amplitude, was needed to estimate any changes in spinal excitability between muscle contraction types. Additional trials were recorded if the coefficient of variation of H-reflex or M-wave amplitudes exceeded 5% across trials. Eight subjects did not have to take additional trials; the remaining seventeen had to take 2.41 ± 1.97 additional trials (mean ± SD), summing all the contraction types.

Finally, two maximal voluntary dorsiflexions (3 s long interspaced by 1-min rest intervals) for each contraction type were performed to record the maximal EMG activity of the antagonist muscle (TA).

### Data analysis

#### Pre-processing

**Figure 6:**
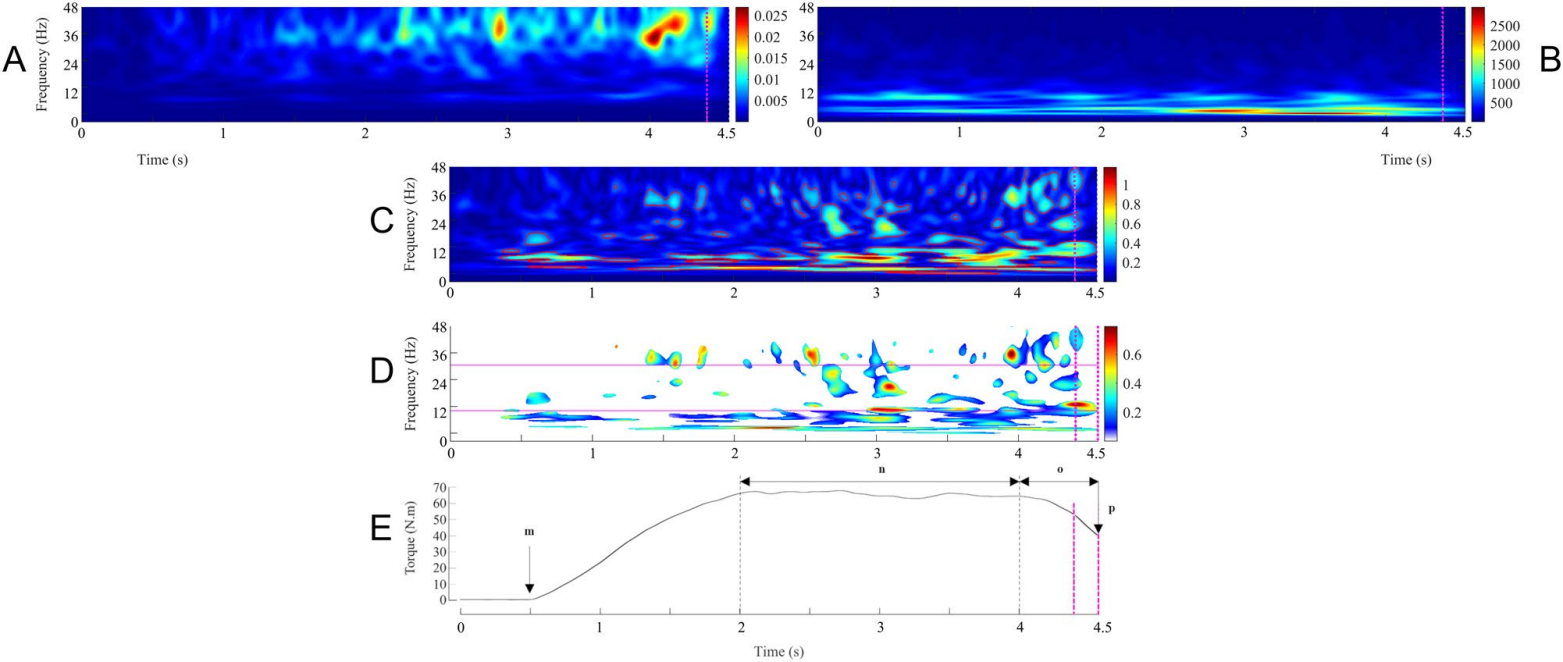
Illustration of the different steps involved in the calculation of time–frequency wavelet-based corticomuscular coherence during shortening contractions, preceded by a period of isometric preactivation, in a typical subject. The pink vertical dashed lines represent the 200 ms window preceding the neurostimulation chosen for the analyse. First row: mean wavelet auto-spectra of the EMG time series from the soleus (**A**) and the Cz EEG (**B**). Second row: wavelet cross-spectrum between the soleus EMG and the Cz EEG time series; the red contours identify the areas in the time–frequency plane where the correlation between the soleus EMG and the Cz EEG signals is statistically significant (**C**). Third row: wavelet magnitude-squared coherence between the soleus EMG and the Cz EEG time series where the correlation between the soleus EMG and the Cz EEG signals is statistically significant. The pink horizontal solid lines represent the [13–32] beta-band. (**D**) In each frequency band (*β*: 13–32 Hz; *γ*: 33–52 Hz), the corticomuscular coherence value was defined as the volume under the magnitude-squared coherence values in the 0.2-s time window of interest preceding the neurostimulation, where the correlation between the soleus EMG and the Cz EEG time series was detected as significant on the wavelet cross-spectrum. Fourth row: mean torque produced during the corresponding contractions (**E**). *G.m.* beginning of the isometric preactivation, *G.n.* 50% EMG SOL isometric preactivation, *G.o.* 50% EMG SOL anisometric contraction, *G.p.* neurostimulation delivery.

On the one hand, the reference-free EEG signal was re-referenced applying the Common Average Reference, calculated by creating an average of all scalp channels and subtracting the resulting signal from each channel to approximate the true voltages over the head^48,49^. The re-referenced EEG signal was then 3–100 Hz bandpass filtered and 49–51 Hz notch filtered with zero-lag, fourth-order Butterworth filters. On the other hand, the EMG signals were downsampled to 1024 Hz (i.e. EEG frequency), and were then 3–100 Hz bandpass filtered and 49–51 Hz notch filtered with zero-lag, fourth-order Butterworth filters. The net joint torque was low-pass filtered at 10 Hz. The continuous torque, EEG and EMG filtered data was epoched in 4.5 s periods of interest, from the contraction onset up to the nerve stimulation (Fig. 6). During isometric contractions, the selected epoch would always be composed of ~ 1.5 s of rest followed by ~ 3 s of ramp and hold isometric contraction. During anisometric contractions, the selected epoch would always be composed of ~ 1 s of rest followed by ~ 3 s of ramp and hold of isometric contraction and then by 0.5 s of anisometric contraction. Trials with large artefacts, such as eye blinks or important contamination by muscle activity, were rejected from the analyses using separate visual inspection of the EEG signal by two different investigators. After rejection, the number of contractions used for further analysis in each contraction type was 7.9 ± 0.1 (mean ± SD) for each subject, with no significant difference between the contraction types (F (2,48) = 2.33; p = 0.11).

#### CMC processing

CMC processing raises methodological concerns that are still discussed in the literature, requiring to clarify the method used in the current study especially regarding to the constraints induced by the quantification of CMC during anisometric contractions.

A first concern is the necessity of EMG signal rectification for CMC analysis. While rectification of the EMG signal is thought to improve the detection of beta-band CMC^50,51^, an increasing number of studies challenges its use. These studies argue that the rectification of a zero-mean oscillatory signal, such as EMG in our case, is a non-linear process that distorts its power spectrum properties^52,53^, and impairs CMC statistical analysis^54^. Since previous studies highlighted that there is no significant difference in CMC magnitude computed with rectified or unrectified EMG signals^55–57^, we opted for non-rectification so as to meet the theoretical demands and uphold the practical justifications for the computation of CMC^42,53^.

A second concern is to deal with the “crucial issue of stationarity” in EMG and EEG time series^58^. Over the last three decades, CMC analysis has been repeatedly investigated in the frequency domain using fast Fourier transformation, with the assumption that electrophysiological signals meet the requirements of stationarity and that the amplitude and phase components of the signals need not be distinguished^59^. However, since non-stationarity is the rule rather than the exception in neural processing, CMC should be studied as a function of time, a constraint that limits the application of frequency analysis^58,60^. The time–frequency analysis, which presents the crucial advantage of taking into account the signals’ non-stationarity—i.e., for how the oscillatory patterns change with time^61^—has thus became a standard method for CMC analysis. Hence, in the current study, CMC was computed in the time–frequency domain to provide a better picture of the spectral variation over time. Amongst several methods devised to compute CMC in the time–frequency domain, it has been shown that the short-time Fourier transform and the wavelet approaches provide a very similar description of the same data^61^. Nevertheless, the wavelet transform easily provides an optimal compromise between the time and frequency resolution^58,60–62^ that is particularly suitable in regard of our experimental design constraints concerning the amplification of the signals’ non-stationarity during anisometric contractions and the relatively small number of trials.

Considering all the above-mentioned methodological concerns, CMC was computed in the time–frequency domain using the Morlet wavelet approach introduced by Bigot et al.; (2011) on Cz EEG (EEG_Cz_) and unrectified EMG_SOL_ or EMG_MG_ signals. The Cz EEG electrode was selected as the electrode of reference, as done in previous studies on plantar flexors CMC, to match the location of the primary motor cortex associated with the lower limb^10,63^. The following CMC processing steps are illustrated for a typical subject in (Fig. 6):

- First step: for each participant, the auto-spectra of the EEG_Cz_ signal and of the EMG_SOL_ or EMG_MG_ signal were computed in the time–frequency domain for all 4.5 s epoched trials performed during a contraction type (n = 7.9 ± 0.1 trials per participant per contraction type) using the WaveCrossSpec Matlab toolbox for wavelet coherence analysis^42^. The wavelet parameters were set to provide optimal identification of EMG and EEG oscillatory activities in different frequency ranges. For the beta band (13–32 Hz), where CMC is associated with voluntary contractions^4,6^, the parameters ‘nvoice’ (scale resolution of wavelets), ‘J1’ (number of scales), and ‘wavenumber’ (Morlet mother wavelet parameter) were, respectively, set to 7, 30, and 10. We also performed a CMC quantification with ‘nvoice’, ‘J1’ and ‘wavenumber’ parameters respectively set to 7, 30, and 17, to provide accurate identification of oscillatory activity in the gamma band (33–52 Hz). Indeed, dynamic contractions have been suggested to increase the amount of the gamma band CMC^33,41^. To cope with the inter-contraction duration variability in the isometric type, that can lead to power spectrum cancelation on both the EMG and the EEG signals, we used the normalization procedure proposed by Fauvet et al. (2019)^64^ to obtain time–frequency auto-spectra of the EEG and the EMG signals.
- Second step: for each participant, the EMG auto-spectra of all 4.5 s epoched trials performed during a contraction type (n = 7.9 ± 0.1 trials per participant per contraction type) were averaged to obtain the mean EMG_SOL_ or EMG_MG_ auto-spectrum of 4.5 s for the corresponding participant and contraction type (Fig. 6A). Using the same procedure on the EEG auto-spectra, the mean EEG_Cz_ auto-spectrum of 4.5 s for the corresponding participant and contraction type was computed (Fig. 6B).
- Third step: the cross-spectrum of the mean EMG_SOL_ or EMG_MG_ auto-spectrum, and the mean EEG_Cz_ auto-spectrum (Fig. 6C), was computed, as well as the magnitude square coherence, using with absolute rigor the equations that govern the calculation of coherence between time-series^1,59^.
- Fourth step: CMC was calculated as the values of magnitude square coherence in the time–frequency regions where the cross-spectrum was significant as detected using the statistical test introduced by Bigot et al. (2011)^26,27^ (Fig. 6D). While the standard test using the wavelet coherence yields many false positive, which might question the interpretability of this test and its level of confidence, the robustness of the method proposed by Bigot et al. (2011) directly comes from this statistical quantification of the coherence, which guarantees that the areas detected as significant in the time–frequency plane correspond to areas where the dependence between the two time-series is "true", or in any case with better accuracy than with the classical time–frequency coherence test.
- Fifth step: CMC volume was finally quantified during the last 200 ms prior to the neurostimulation onset for both the SOL and the MG in the beta-band (β-CMC_SOL-Cz_ and β-CMC_MG-Cz_, respectively) and in the gamma-band (γ-CMC_SOL-Cz_ and γ-CMC_MG-Cz_, respectively).

#### H-reflex, M-wave and EMG activity

For each contraction type, the mean peak-to-peak amplitude of SOL and MG H-reflexes (H_sup_) and M-waves (M_sup_) for all trials was calculated. The corresponding H_sup_/M_sup_ ratios were then computed to investigate the modulation in spinal excitability. The amplitude of the submaximal M-wave evoked at H_sup_ (MH_sup_) was measured to control the stability of the stimulus intensity (MH_sup_/M_sup_, ratios). Since the stimulation intensities were not optimized for the MG, the corresponding data for EMG activity and the evoked potentials was only analysed in a subgroup of subjects (n = 14), in which simulation conditions allowed the study of spinal excitability as complementary results.

EMG_SOL_ and EMG_MG_ activities were quantified with RMS values of the EMG signal over a 200-ms period prior to the stimulation and were normalized to the mean amplitude of the M_sup_ (RMS/M_sup_) obtained during the same contraction type. For each contraction type, the mean value over all trials was considered to represent the EMG activity for both the SOL and the MG. During the same period of time, the TA RMS prior to the stimulation was also analysed for the three contraction types. To quantify the level of coactivation during the isometric contractions, the TA RMS was expressed as a fraction of its value determined during MVC dorsiflexions. To quantify coactivation during shortening trials, the TA RMS obtained during shortening plantar flexions was normalized to its value determined during maximal voluntary lengthening dorsiflexions whereas to quantify coactivation during lengthening trials, the TA RMS recorded during lengthening plantar flexions was normalized to its value measured during maximal voluntary shortening dorsiflexions^65^. For each experiment, a mean value of coactivation was computed over all trials for each contraction type.

Additionally, to quantify the contraction type-related changes in the EMG mean frequency for both the SOL and the MG, we determined the sum of product of the power spectrum and the frequency divided by the total sum of the power spectrum for both the SOL and the MG (EMG SOL mean frequency and EMG MG mean frequency, respectively). Both SOL and MG EMG mean frequencies were calculated, for each participant and each contraction type, from the same EMG’s respective mean auto-spectrum computed with the wavelet transform during the CMC processing.

### Statistical analysis

All the data is presented as mean ± standard deviation (SD). The normality and sphericity of data were verified using the Kolmogorov–Smirnov test and the Mauchly’s W test, respectively. Separate ANOVAs with repeated measures were used to test differences between contraction types (isometric vs. shortening vs. lengthening) for the plantar flexors torque, RMS/M_sup_, co-activation, EMG mean frequency, and for both the SOL and the MG the H_sup_/M_sup_, M_sup_ and MH_sup_/M_sup_. Separate two-factors (muscle (SOL vs MG) × contraction type (isometric vs. shortening vs. lengthening)) ANOVAs with repeated measures on the muscle and the contraction type were used to compare β-CMC and γ-CMC. When a main effect or an interaction was found, a post hoc analysis was made using a Scheffe test. The correlation between β-CMC and H_sup_/M_sup_ in the SOL was tested using a linear regression analysis (Pearson’s product–moment correlation). The difference of β-CMC between isometric and lengthening contractions (ΔCMC) was correlated to that of H_sup_/M_sup_ (ΔH_sup_/M_sup_) using also a linear regression analysis for the SOL. Significance was set at p < 0.05. ANOVAs’ results are presented along with partial eta squared (η^2^), and the effect size measurement Cohen’s f (f). Statistical analyses were performed using R (R, version 3.5.1, 2018-07-02).

## Data Availability

The data that support the findings of this study is available on request from the corresponding author. The data is not publicly available due to privacy or ethical restrictions.
